# Policy experimentation and innovation as a response to complexity in China’s management of health reforms

**DOI:** 10.1186/s12992-017-0277-x

**Published:** 2017-08-03

**Authors:** Lewis Husain

**Affiliations:** Institute of Development Studies, Library Road, Brighton, BN1 9RE UK

**Keywords:** Global health, Scale up, Complex adaptive systems, China, Health reform

## Abstract

There are increasing criticisms of dominant models for scaling up health systems in developing countries and a recognition that approaches are needed that better take into account the complexity of health interventions. Since Reform and Opening in the late 1970s, Chinese government has managed complex, rapid and intersecting reforms across many policy areas. As with reforms in other policy areas, reform of the health system has been through a process of trial and error. There is increasing understanding of the importance of policy experimentation and innovation in many of China’s reforms; this article argues that these processes have been important in rebuilding China’s health system. While China’s current system still has many problems, progress is being made in developing a functioning system able to ensure broad population access. The article analyses Chinese thinking on policy experimentation and innovation and their use in management of complex reforms. It argues that China’s management of reform allows space for policy tailoring and innovation by sub-national governments under a broad agreement over the ends of reform, and that shared understandings of policy innovation, alongside informational infrastructures for the systemic propagation and codification of useful practices, provide a framework for managing change in complex environments and under conditions of uncertainty in which ‘what works’ is not knowable in advance. The article situates China’s use of experimentation and innovation in management of health system reform in relation to recent literature which applies complex systems thinking to global health, and concludes that there are lessons to be learnt from China’s approaches to managing complexity in development of health systems for the benefit of the poor.

## Background

Scale up of health interventions in low- and middle-income countries (LMICs) is increasingly a priority for development agencies and policy makers. Attention to scale up responds to the need to expand coverage of interventions targeting specific diseases, roll out interventions addressing specific population groups, and strengthen health systems in LMICs for the achievement of broad health goals [1, 2]. One of the most obvious attempts at global health scale up is the Millennium Development Goals (MDGs). Success of many programmes, and success in reaching the health MDGs, has been uneven [3]. This is increasing policy and research attention to how health interventions can be ‘scaled up’ in LMICs. Recent research is shifting attention from supposedly universally-applicable best practices towards more context-sensitive implementation processes [4] and learning that can inform better programming and scale up. Recent research has argued that health systems, especially in LMICs, should be viewed as complex systems, and this has implications for how scale up can be achieved [5]. This paper discusses the case of China. While China’s health system remains a work in progress, substantial progress has been made in developing health systems with broad population coverage, addressing communicable diseases, improving maternal and child health and in meeting MDG targets. China is notable for discussions of scale up: substantial discretion in policy implementation is encompassed within a nominally unitary political system, and many reforms incorporate experimentation and innovation, which can contribute to systemic learning and help inform policy development. This is part of a discernible Chinese government ‘work style’ [6]. This paper argues that this has advantages for scale up of health interventions in a huge country in which reforms must be applicable across a wide range of places, under conditions of complexity, and with limited resources and capacity, and that China’s domestic experience of using experimentation in health system reforms has relevance for other countries engaged in complex systemic reforms, including health system reforms. In addition, as China increasingly becomes a development actor, and engages in overseas health programming, it is likely to draw on its own reform experience in assisting other countries. As Chinese agencies increasingly engage in overseas health programming, this will provide a test of the broader applicability of Chinese-style experimental policy processes and their adaptation to new environments.

### Global health and challenges of scaling up

The term ‘scale up’ has increasingly been used to frame a set of challenges to implementing health interventions and developing functioning health systems. In its core meaning, it refers to increasing the “coverage of health interventions that have been tested in pilot and experimental projects in order to benefit more people and support policy and programme development at a large or national scale”, though it is often used more broadly to mean increasing the geographical or population coverage of an intervention, or increasing resources committed to an intervention [7]. The language of scaling up has also been applied in global health debates to implementation in LMICs of interventions shown to be successful elsewhere or to health systems strengthening. Paina and Peters [5] exemplify this use, including delivery at scale of child health interventions, promoting roll-out of anti-retroviral therapy, and expanding access to core packages of health services in LMICs as part of the Millennium Development Goals (MDGs). Despite increased funding, and attention from policy makers and practitioners, attempts at scale up in LMICs have had varied success, as shown by limited success of interventions that have been shown to be effective elsewhere [8] and failure of many countries to achieve the health MDGs [5].

Recent research has argued that global health initiatives predominantly assume that technical interventions tested in one place can be replicated at scale in different contexts, and that most research on such initiatives has been results-focused (e.g. focusing on coverage rates and mortality) rather than on processes of scaling up specific interventions in new contexts [7, 9, 10]. Simultaneously, there is increasing attention to the importance of context in implementation of health interventions [11, 12], realization that there are no universally applicable approaches to scale up of health interventions [1, 2, 11] and that interventions in different contexts, though called by the same name, may in fact differ substantially [2]. This is leading to a realization that the *process* of implementation (scale up) itself may be as important as the strategy to be implemented, and increasing attention to ‘implementation research’ – research on processes of implementation and how these affect programmes [4]. This presents a challenge to mainstream ideas of scale up in its various forms, including how to apply interventions that have been shown to be effective elsewhere, and how to scale up experimental interventions and pilots to achieve greater population coverage and health benefits. Researchers have addressed the challenges of scale up in a range of ways. I review prominent analyses, and argue: that challenges of scale up are not simply challenges of overcoming certain easily identifiable constraints located within health systems, but that reforms to health systems, especially in rapidly developing countries, are embedded in, and involve, broader social, economic and institutional reforms; and that many health system interventions, especially when considered in context, show many features of ‘complex systems’. Reforming such systems requires approaches to change management that foster innovation, adaptation and learning.

*Constraints to scale up*. Recent research has identified financial, infrastructure, human resource, and medical supply constraints to scale up, as well as constraints related to policy, institutional and organisational structures and processes. Hanson et al. [8] create a typology of constraints, identifying those which are comparatively easily overcome through increased spending, and those which are less so (e.g. constraints linked to governance and government capacity), and which require other approaches.^1^ Gericke et al. [13] argue that there is insufficient consideration of the technical challenge of health interventions in low resource contexts, and that non-financial resources are major constraints to scaling up. Other analyses discuss alternative approaches to increasing coverage, intended to side-step constraints to scale up through use of vertical or single-issue programmes, which can be comparatively isolated from other elements of the health system, delivery through non-governmental organisations [7], and through partnership with the private sector [14].

### Fostering emergence in complex systems

An increasing number of analyses use complex adaptive systems (CAS) theories to reframe how scale up of health interventions in LMICs should be understood, and critique ‘blueprint’ implementation approaches [5]. CAS deals analytically with systems in which actions of any one agent, or group of agents, change the environment of other agents, leading to adaptive behaviours, self-organisation and learning, and cascade effects with unpredictable and emergent order [15]. This challenges linear understandings of interventions and scale up [16, 17], compounded when health systems are considered in political, economic, social and institutional context [10], where the challenge is not to specify an end point, but to find a path to it [18]. Finding that path, where ‘what works’ is not (fully) known in advance, involves understanding and working through complex systems. This section argues for placing *emergence* at the centre of analysis of how to intervene in complex systems.

Complex systems generate emergent order, “when smaller entities on their own jointly contribute to organized behaviours as a collective, resulting in the whole being greater and more complex than the sum of the parts” [5]. This is important because, as researchers, policy makers or implementers, most phenomena we are interested in are emergent system properties, whether that be a functioning economy in which multiple firms interact within regulatory structures and with consumers to provide goods and services [19], or a health system, in which innumerable elements come together to provide appropriate and affordable care. In order to accelerate and shape the development of a functioning and equitable health system, we must understand how the environment can be made conducive for the emergence of desirable self-organising complexity [20]. This means asking process questions (‘how should we learn what works in the current situation?’), as well as trying to define best practice (‘what works?’). It requires willingness to experiment and innovate, and to accept that intervening in complex systems is challenging, often does not produce hoped-for outcomes, and requires that interventions (whether discrete programmes or policy-driven systemic reforms) be treated as learning processes, rather than implementation of fixed ‘blueprints’.

The remainder of this section summarises points from analyses of intervening in CASs to support discussion in the following section of how China has attempted to stimulate, and learn from, policy innovation, and the relevance of this to managing challenging reforms in large and rapidly-changing complex systems.

The following table synthesises a number of approaches drawn from health systems and development studies literatures on intervening in complex systems. This brings out commonalities in both literatures around the need to:

- Scope system and dynamics and identify points for intervention
- Experiment and encourage innovation and policy pluralism
- Foster methodological pragmatism and appropriate policy solutions
- Screen for, and learn from, policy innovations and useful practices
- Promote rapid learning and diffusion of useful practices, but allow flexibility during scale up

The table groups main points from a range of studies that analyse approaches to intervening in complex systems. In line with the key theoretical point of this section, these points are oriented towards an analysis of approaches for fostering the emergence of order in complex systems where highly-directed planning is of limited value. The following points summarise commonalities arising from the analysis presented in Table 1. This analysis is used to inform the discussion of Chinese approaches below.

1. The assumption underlying these approaches is that planning is of limited use in complex systems, and that desirable outcomes must be fostered. This says nothing about the degree of complexity of any given system, but is a statement of principle. Not all interventions should be considered complex in this sense, and different interventions therefore present different challenges.^2^

2. There is substantial consensus on the importance of creating the conditions to allow change and to stimulate innovation, rather than planning. A range of incentive structures will be important, and will likely be context-dependent.^3^ Many experiments and innovations fail, and this requires development of cultures of tolerance for policy or implementation failure, and developing incentive systems that are goal based, rather than task-based. ‘Isomorphic mimicry’ [21] refers to the danger of developing institutions that look like those in, say, developed countries, but which are hollow or not useful due to poor contextual fit.^4^

3. Analyses recognise the importance of finding institutions that fit a given context, even if these are less technically efficient than theoretically-known best practices, rather than trying to impose best practice solutions. This makes sense if complex systems are judged to be evolutionary – ensuring adaptation over time is more important than maximising efficiency in the short term [22].
4. ‘Positive deviance’ encapsulates a simple idea – in any given cohort, there are likely to be better-than-average practices. These deserve attention from researchers and policy makers looking for ways to foster positive changes.^5^ Such practices may be ‘naturally occurring’ and precede intervention, or they may result from intervention where the parameters are wide enough to foster a range of approaches and innovation of the part of implementing units.
5. Interventions in complex systems function as probes – they generate information on the nature and functioning of the system that can be used to inform subsequent intervention [23]. A ‘failed’ intervention may therefore be useful for the information it provides, though failing reforms should be discontinued where appropriate.

6. Fostering dispersed innovation is methodologically distinct from managed experimentation, though the aims are similar – how to capture dispersed initiative and uncover useful ways of working? Screening useful practices from a mass of policy experience requires developing fitness functions that can promote good (‘fit’) practices and discourage inappropriate ones [24]. Screening may be simple (e.g. using trusted research institutions to carry out evaluations of emerging practices), or more decentred and organic.
7. In evolutionary systems, when promoting emulation of policy solutions, replicability is more important than technical efficiency (this mirrors point 3, above). Enforcing standardisation during roll out may have the same kinds of drawbacks as enforcing one-size-fits-all policy implementation.
8. None of the analyses discussed here specifically addresses the normative question of what should be considered desirable or takes that into account in discussion of complex systems and fostering emergence, though most analyses have an implicit value orientation. I return to this below.

**Table 1:** Key features of analyses of intervening in complex systems

Requirements/approaches	Core principles
1. Scope system and dynamics; Identify stakeholders and critical change points for intervention	- Attempt to identify key stakeholders, networks and ‘critical points for change’ in advance of reforms [5], but be ready to learn rapidly about system dynamics and adjust as reforms are implemented - Make use of tools such as scenario planning and agent-based models to identify CAS phenomena, attempt to anticipate changes and guide interventions [5], but be prepared to adapt - Engage multiple stakeholder groups in conceptualising and planning interventions to help to capture dispersed understandings of system complexity [68]
2. Experiment and encourage innovation and policy pluralism	- ‘Plan for unpredictability’: create conditions for change, rather than trying to directly engineer change; allow flexible implementation by implementing units (sub-national governments, departments and others) over rigid adherence to initially-conceived plans [5] - Use experimental policy frameworks to stimulate systemic innovation; allow and encourage trial and error by implementing units and tolerate implementation failure within reasonable bounds [23] - Use incentive systems that incentivise achievement of agreed goals (however these are achieved) over carrying out of specific processes as a way to encourage pragmatic problem solving and foster emergence of multiple policy solutions [23] - Directly experiment and contrast multiple approaches where needed and/or where fostering dispersed innovation is failing to find workable policy solutions [69]
3. Foster methodological pragmatism and appropriate policy solutions	- Encourage methodological pragmatism and pluralism in problem solving by implementing units over preconceived approaches (fit solutions to problems; not problems to solutions) [70]; recognise that there may be multiple different solutions to a given problem or multiple practices; accept diversity in policy solutions adopted by implementing units - Look for policy solutions that are contextually appropriate and show ‘fit’ with the environment, rather than getting hung up on ‘best practice’ solutions; recognise that apparently technically second best approaches may function as ‘stepping-stone’ policies, may promote system adaptation, and can be improved on in subsequent rounds of reform [71] - Avoid ‘isomorphic mimicry’, or practices that mimic supposed best practice, but have little substance [70]
4. Screen for, and learn from, policy innovations and useful practices	- Accept that in a CAS, solutions to problems must be discovered, and that there are limits to ex ante design [72]; decrease reliance on planning and ex-ante analysis*,* and increase monitoring, learning and adaptation during experimentation-implementation [73] - Incorporate multiple types of evaluation to capitalise on emerging understanding of intervention processes and effects during roll out [68] - Screen for ‘positive deviance’, or naturally occurring useful practices and emerging innovations, and encourage dissemination of these through appropriate ‘fitness functions’ that can promote adaptive learning while discouraging inappropriate practices - Promote appropriate and replicable (though not necessarily optimally efficient) policy innovations over optimally efficient policy solutions that will be hard to replicate [74, 75]
5. Promote rapid learning and diffusion of useful practices, but allow flexibility during scale up	- Carry out iterative programming / reforms; promote rapid learning, and ‘fail fast’ – directly terminate failing or inappropriate practices or allow implementing units a degree of discretion over this [23] - Make use of diagnostic tools to analyse staging of reforms and think through the process of removal successive binding constraints to reform [21]; use such tools to help sequence reforms, but recognise that variation in starting points and conditions will lead to implementing jurisdictions or units simultaneously occupying different stages of reform trajectories - Proactively propagate and support useful policy innovations, but be wary of imposing one-size-fits-all policy solutions during scale up [10]

**Table 2:** Indicative typology of China’s experimental health policy processes

Indicative type	Salient features
Type I: Managed piloting/policy trialling	- Direct experimentation, allowing trialling of targeted interventions in which pilots are relatively closely managed with the intention of trialling specific approaches to defined policy problem; local governments have a relatively low degree of discretion - Technical support to implementing units is often provided by research institutes, academics, and/or international agencies; local governments retain discretion in concrete management approaches adopted and in timing, etc., in an attempt to find approaches with contextual fit - Pilots may be in advance of the national (or provincial) policy agenda, and have an agenda setting function, or may fall within existing policy frameworks and form part of ongoing reforms and may provide lessons of supra-local or systemic significance - Screening and learning: scale up of practices deemed useful may or may not take place; may be directed by higher levels of government (frequently through one size doesn’t fit all scale up) or may be relatively organic
Type II: Experimental policy frameworks; local government purposive reforms	- Framework policy is set by central or provincial government, giving local governments or other implementing units limited discretion between relatively defined implementation choices; leads to multiple practices - Implementing counties often have little expert support or technical assistance, though better-resourced jurisdictions may have support from national or sub-national research institutions, or occasionally external TA through international programmes; space for pragmatic problem solving and emergence of ‘appropriate’ approaches with contextual fit - Local government reforms fall within the ‘implementation’ phase of the policy cycle; may provide lessons of supra-local or systemic significance - Screening and learning: as above
Type III: Open policy frameworks; local government adaptive innovation and learning by doing	- ‘Open’ policy frameworks are used by central government, allowing space for broad local discretion in implementation and learning by doing and emergence of multiple practices^11^ - Often little expert support – as above; space for pragmatic problem solving and emergence of ‘appropriate’ approaches with contextual fit - Local government innovation falls within the ‘implementation’ phase of the policy cycle; may provide lessons of supra-local or systemic significance - Screening and learning: as above
Type IV: Decentralised implementation; range of policy practices	- Decentralised policy making in the absence of national standardisation can produce a range of policy practices - Often little expert support – as above; space for pragmatic problem solving and emergence of ‘appropriate’ approaches with contextual fit - May fall in multiple phases of policy cycle; may provide lessons of supra-local or systemic significance - Screening and learning: as above

^11^Open’ policy frameworks deliberately allow implementers discretion in the co-construction of reforms [76]. Much Chinese policy is of this type, though by no means all local implementers are proactive innovators or engaged in learning by doing, and much foot dragging [77] and mis-communication also [78] exist, while financial resources and prior policy design may be inadequate [42].

The points presented here derive from a substantial, but scattered literature, and could easily be extended; however, they provide a starting point for analysis of a distinct Chinese approach to health reforms in the next section.

## Understanding experimentation in China’s health reforms

Since the beginning of reforms in the late 1970s, and the move away from a state-dominated and planned economy, China has been transformed from a poor country, in which incomes of most of the population stood below the global median, to a middle-income country, raising hundreds of millions out of poverty in the process [25]. Latterly, it has actively developed social protection systems, including rural and urban health financing schemes, pensions for rural and urban populations, and others. China’s approach to reform has been government-led and unorthodox. The speed of development, and the complexity and interconnectedness of reforms have led many to talk of a specific ‘Chinese model’ of development [26]. Experimental policy making and policy innovation are part of the policy toolbox, though most analyses have discussed experimental policy making in economic reform and similar areas, rather than health or social policy. This section argues that China’s health system development has relied substantially on a range of approaches to policy making, implementation, and policy learning that can be broadly classified as experimental. The discussion that follows is not exhaustive: health reforms have been underway for more than 20 years, and reforms are ongoing both in the health system itself, and in the larger institutional-bureaucratic system. It is also not to say that Chinese approaches are optimally efficient, or that they are necessarily effective (or are used well), but this section argues that China’s approaches (China displays not one form of experimental policy making, but a range of approaches that fall under this umbrella) provide real-world examples of the use of experimental reform management techniques in reforming complex systems, and that these deserve attention from researchers and policy makers concerned with scale up of health interventions.

### Complexity in China’s health reforms

As argued above, health systems should be analysed in their social, economic and institutional context. Seen in this way, Chinese health system reforms show a high degree of complexity. While this is hard to capture overall, at least the following points are highly salient:

- The health ministry sits at the apex of a hierarchical bureaucratic system and makes policies that guide the actions of multiple tiers of lower level government, but health policy exists within a broad ecology of policy making involving many institutional actors and interests.^6^ Policy making responsibility is distributed, and many policies with direct relevance for health agencies, healthcare providers, etc., are the responsibility of other parts of government. Actions of a range of other government and institutional actors may have an impact on those of health agencies. Central government coordination groups are often set up at times of intense reform activity to coordinate ministry interests and link mandates.

- Central government policy must be sufficiently broad to be implementable in jurisdictions (principally cities and rural counties, henceforth: counties)^7^ that vary across many parameters, including levels of economic development, financial and human resources, population health, geographical, environmental, socio-cultural and spatial factors. Equally, counties’ bureaucratic and institutional structures differ, reflecting naturally occurring variation in the political economy, and the legacy of past restructuring processes (both within and outside the health system) that have been implemented differently, or to different degrees, in different places. The importance of non-state actors (including civil society and commercial interests) differs by place.
- The number of counties and the range of parameters across which variation may occur create great information asymmetry for central ministries and even provincial governments, and data able to inform intervention are often unavailable, or are limited, before the launch of a given reform. The results of interventions across a range of counties with different conditions and starting points are hard to foresee and the possibility of divergent outcomes in different counties, or over time, is high. The interactions between reforms in the health system and other social, administrative, economic, and environmental systems are harder to predict, especially given ongoing and rapid change in almost all such systems, an increasing amount of which falls outside the direct influence of government, as the society and economy are liberalised. Rapid change in all these areas creates challenges for predicting the effects of any given intervention.

Since the early 2000s, China has embarked on a process of rebuilding a functioning health system, following the partial dismantling of its pre-reform system, increasing marketisation of many health services, and worsening access and equity [27, 28]. Since the early 2000s, waves of reforms have focused on a wide range of areas, including launching of rural and urban health insurance programmes to underpin population health seeking, increasing government health spending and, under the aegis of the 2009 new health reform package, expanding insurance coverage, establishing a national essential medicines system, improving primary care and improving availability of public health services, and piloting public hospital reforms [29]. The above changes have been accompanied by vast transformations of China’s economy, infrastructure, population, and society.

As above, the starting point of much scale up literature is an intervention that is thought to merit application at greater scale, and this literature increasingly points to the importance of context and an understanding of complexity in ensuring successful roll out. On any possible interpretation, building a functioning health system in China requires dealing with complex, high speed and intersecting reforms across a wide geographic area in which the starting points of implementing counties are hard to know, and in which the results of reforms are hard to accurately predict. The following sections discuss Chinese thinking about experimental policy development, and a range of experimental management techniques visible in China’s health reforms.

### Central control and local discretion

In China, local experimentation as a tool for policy development dates to before the establishment of the People’s Republic of China (PRC) in 1949, and was pioneered by Chinese communist party (CCP) cadres as a way to deal with a range of priority issues, including land reform and organising agricultural production. For the CCP, this was a pragmatic approach to reforms under conditions of scarcity of resources and expertise and in an environment characterised by extreme variation in conditions. Approaches trialled in the 1920s and 30s were progressively codified as party/government working methods and as an identifiable administrative vocabulary of reform management [30]. Such pragmatic approaches to reform management have existed in tension with more command-oriented approaches over the history of the PRC, but have been frequently been stressed in China’s ‘reform’ era, starting in the 1970s, through use of experimental zones and experimental policies. Such deliberate experimentalism coexists with increasing discretion within the government system through decentralisation of administrative authority and creating incentives for local governments to pursue reforms (such as in economic development) that have system-wide benefits [31]. A number of factors underlie Chinese thinking on this, not least the difficulty of adopting one-size-fits-all solutions in a country of continental size and encompassing very great variation. Inevitably, the range of dynamics created by policy approaches that include direct experimentation as well as decentralisation mean that a range of patterns of learning exist, from (in some cases) directed scale up of relatively managed experiments to more organic dissemination of scattergun innovation processes tied to decentralisation [32].

In many policy areas, central government leaves substantial space to sub-national governments within an overall policy framework and direction set by central government, creating a ‘paradox’ of central control and sub-national space for discretion and initiative: while central government controls the policy agenda, the personnel system, much resource allocation, and approval for large projects, sub-national units have discretion and space for local initiative, which can support policy innovation [33]. This is frequently rationalised with reference to common understandings of the ‘spirit’ of policy:

“China is a unitary polity. Sub-national government must obey central government, and must carry out reform under the unified arrangement and direction of the centre. [However] central institutions and policy give local government a lot of space for exercising initiative. Acting according to the requirements of the centre, and under the unified leadership of the centre, doesn’t mean blindly or mechanically acting according to the instructions of superior levels [of government]. Rather, each place can […] in line with their local conditions, experiment boldly according to the spirit of central [policy]” [34].

This framing of local government flexible implementation and innovation within the overall spirit of central policy pre-dates the establishment of the PRC. The paradigmatic statement of this comes from Mao Zedong:

“legislative powers are all vested in the central authorities. But, provided that the policies of the central authorities are not violated, the local authorities may work out rules, regulations and measures in the light of their specific conditions and the needs of their work […] We want both unity and particularity. [I]t is imperative to have a strong and unified central leadership and unified planning and discipline throughout the country […] At the same time, it is essential to bring the initiative of the local authorities into full play and let each locality enjoy the particularity suited to its local conditions” [35].

This framing is tied to policy discourses which emphasise the importance of ‘implementing according to local conditions’ (*yin di zhi yi*), and the impossibility of adopting ‘one size fits all’ (*yi dao qie*) policy that can be summarised as a “deep-seated one-size-does-not-fit-all pragmatism” [36]. Central leaders and policy makers frequently express rhetorical commitment to sub-national policy adaptation, experimentation and innovation,^8^ underpinned by a policy discourse that allows signalling of emergent practices. This discourse of local particularity is shared by many actors within the policy community, including sub-national officials, think tank researchers and academics,^9^ and one aspect of China’s reform process has been to create norms of tolerance of variant sub-national policy practices that can allow initiative and risk taking by local governments for systemic benefit [31].

### What does experimentation look like?

Multiple forms of activity should be considered under the umbrella of ‘experimentation’ in Chinese policy development. According to Heilmann [37, 38], Chinese central government supports managed pilots for the exploration of novel policy options intended to support central decision making, but other analyses have shown widespread policy ‘tinkering’, in which sub-national governments carry out broad low-level experimentation on a range of issues under loose policy frameworks [39]. In most cases, experimentation is not controlled piloting, but distributed problem solving under overarching national policy frameworks, often signalled by terms such as ‘innovation’ [40, 41]. This fits closely with the typology of approaches to intervening in complex systems to promote the emergence of desirable order discussed in Fostering emergence in complex systems and Table 1. Experimental policy making of some form exists in many policy areas, including health programming [36, 42–44].

This section develops an indicative typology of forms of experimental policy making, implementation and innovation. This is a heuristic device, whose aim is to indicate the breadth of activity that should be considered under this rubric, to show commonalities with the analysis, above, of programming under conditions of complexity, and to raise the question of the relevance of such approaches for researchers and policy makers concerned with scale up of health interventions (Table 2).

A brief overview of development of China’s rural health insurance scheme, the New Cooperative Medical Scheme (NCMS), illustrates a number of these aspects. A section on Important features and limitations, below, extracts distinctive features from this narrative and frames them in the context of the analysis provided in the section on fostering emergence in complex systems.

- Following the decline and collapse of China’s pre-reform rural health insurance, a number of pilots were run in the 1990s and onwards, both managed pilots, involving a substantial degree of expert design or oversight **[Type I]** [45], and pilots in which sub-national jurisdictions were granted a degree of autonomy to decide their own reforms **[Type III]** [46]. Pilots often had support from Chinese and foreign researchers and international agencies, aiming to provide a model for a new insurance scheme. While lessons were learned about possible modes of structuring a new rural insurance scheme, and system dynamics, these failed to have substantial policy impact until the opening of a policy window in the early 2000s [47]. Lessons from these early pilots were, to an extent, taken into account in establishing a national ‘pilot’ scheme [36, 48].

- The national pilot scheme initially gave around 300 local governments a range of implementation choices [49, 50] in scheme design **[Type II]**, though much sub-national management was unscripted trial and error within the overall parameters of the scheme **[Type III]**. Incomplete dismantling of the previous rural health insurance programme from the 1980s onwards meant that some counties retained insurance schemes **[Type IV]**, while variation in economic, institutional and political economic factors provided varying starting points for reforms in different locations **[Type IV]**.
- This range of experimental processes produced a wide range of approaches and practices of varying degrees of local and supra-local usefulness, as well as some approaches deemed illegitimate. Efforts were made to learn from this scattergun implementation experience, to disseminate lessons from it in reports and guidelines [51, 52], and to use it in codification of policy. In many areas, sub-national governments (counties and provinces) made concrete policy which was then learnt from and/or codified in provincial or national regulations, in a kind of policy ‘crowd sourcing’ [44].
- Even following roll out, much scheme management remained under-institutionalised [53], and sub-national governments engaged in much learning by doing **[Type III]**, figuring out how to manage a complex insurance scheme, and to develop suitable management arrangements that are contextually appropriate as they went along, often with little support. Such local experimentation / innovation underpins ongoing system reform and resilience, is often widely reported, and may be the focus of debate within the policy community, but may or may not have much policy impact or be codified [54].
- Faced with specific management problems, national and provincial policy makers, research institutes and international agencies commissioned or carried out managed pilots **[Type I]** or made use of experimental policy frameworks **[Type II]** in an attempt to develop policy models with broad applicability, for example in developing functioning provider payment systems [55, 56]. Some such models were propagated widely, but policy impact is often limited and/or hard to show.

- Sub-national governments carried out a range of reforms within the overall parameters of the scheme, whether through proactive policy entrepreneurship or through pressure to implement the scheme **[Type III]**, exploring different scheme management problems, including fundraising approaches, cost control measures, payment reforms, linking rural and urban schemes, extending the scheme to rural-to-urban migrants, and similar. This scattergun approach produces a wide range of results, of varying degrees of usefulness. National, city and provincial policy makers, as well as academics and researchers, made use of a range of methods (site visits, meetings, evaluations, etc.) to identify and propagate potentially useful practices – a form of screening for, and learning from, positive deviance – and there is often substantial debate over reforms and specific practices, though propagation is often limited.

### Important features (and limitations)

The above is a sketch of a highly complex reform process run across almost three thousand implementing counties over the course of approximately two decades, from small scale managed pilots preceding national policy backing, to the launch of a loosely-articulated piloting process in the early 2000s, nationwide roll out, and ongoing adaptive management, problem solving, learning by doing, and increasing codification over time, including linking of the rural health insurance scheme with urban insurance schemes [57].^10^ The case presented here illustrates very many, if not most of, the approaches to managing for the emergence of order in complex systems described from the literature in Fostering emergence in complex systems. The following features are striking:

- Central government controls the overall policy agenda and direction of reform, sets implementation targets for local governments (frontline implementers of the national scheme), and has oversight of scheme financing. Coexisting with this control, many implementation parameters are only broadly defined, and sub-national governments act as problem solvers and change managers, rather than straightforward implementers of policy. Central government encourages and tolerates a wide range of policy practices. Variation between implementing counties is substantial, and a wide range of policy practices exists at any one time.

- Sub-national reforms frequently ‘die’ or are discontinued, in their place of origin, whether because they are deemed unsuccessful, or because of changes in local leadership, incentives or conditions [58]. On the other hand, some sub-national reforms show patterns of iterative learning and deepening [59].

- While variation creates substantial information asymmetry and difficulties for learning, multiple patterns of policy learning exist, from ‘vertical’ codification of policy practices deemed useful in provincial or national policy, through to relatively organic spread through a range of ‘informational infrastructures’ [60], including meetings, trainings, organised site visits, exchange of documentation, etc. Reforms are accompanied by substantial information flows, through policies, ministry bulletins, media, etc. Policy champions and trusted intermediaries, such as government research institutes, may be important in promoting certain sub-national practices.
- Variation across jurisdictions can occur along multiple dimension; scale up inevitably encounters multiple contexts and requires ongoing policy adaptation. Codification of diverse policy practices often occurs late in the policy cycle, maintaining space for sub-national flexibility and one-size-doesn’t-fit-all scale up. Even where codification occurs, reforms are often ‘under-institutionalised’ [53].
- There are many criticisms of such approaches to policy development [61]. This is not an optimally efficient approach to policy development: many pilots fail to have substantial impact, while many policy innovations fall by the wayside or fail to be propagated [50]. Specific policy innovations are rarely ‘best practice’, but the system can produce ‘appropriate’ solutions that show contextual fit, and underpin ongoing system adaptation [44]. Overall, there is space for policy learning to take place and for system adaptation over time.

As discussed above, the above dynamics are visible in a range of reform areas, in health and elsewhere, and a substantial body of Chinese analysis is devoted to profiling potentially useful or important sub-national reforms.

## Conclusions

This paper has discussed scale up in global health from the point of view of complex adaptive systems and has synthesised strategies for engaging in complex systems. It has argued that emergence should be the key feature of interest for policy- and intervention-focused researchers, and that the question of how to foster desirable emergent system properties is core to a ‘complex systems approach’ to health system change, and hence to scale up in global health in complex contexts. The paper argues that China’s management of change shows many features that the literature tells us are necessary to management of change in complex systems, and presents a case study showing the operation of these in one area of China’s ongoing health reforms.

Use of China as a case does not imply that approaches such as these to health system development are a panacea; they are not. China’s reform management, including management of experimental reforms and promotion of innovation, suffers from many problems, not least low efficiency and relatively limited popular accountability. The particular ‘implementation conjuncture’ of sub-national governments as well as, in many cases, limited local government capacity and finances, often influence sub-national behaviour [44]. Though this is by no means unique to China, reforms which challenge the interests of powerful groups (such as hospitals) [24], or which pit one government ministry against another, are likely to be harder to implement – and are likely to be less promising areas for fostering experimentation and innovation in the service of complex reforms – than those that don’t. Overall, evolutionary approaches to economic (or other) development caution that allocative efficiency may be low in such systems, but that this may be a price to pay for adaptation over time [32]. While China’s health system continues to have many problems, progress is being made in ensuring broad population access and reforms continue in many areas.

China’s adoption of pragmatic, experimental approaches to reform management appears to have been the result of a specific conjuncture, and of the difficulty of managing a country of China’s size and heterogeneity. These approaches have been developed over time and, to an extent at least, codified. While the institutional foundations of China’s experimental-innovative approach to reform are receiving increasing attention [6, 38], there remain questions about its institutional basis. This article has shown how Chinese health system reform processes show a lot of the features stressed in the literature for managing change in complex systems through fostering dispersed experimentation and innovation, screening and learning from decentralised experimental processes, and through use of appropriate transitional institutions in ongoing and evolutionary reform processes. Globally, there remain many questions about how concretely to manage reforms in complex systems, particularly how to ‘manage for emergence’. Many debates on the fostering and propagation of innovation in social policy originate in developed countries [62–64], and it is increasingly clear that institutions are very context-specific, making necessary context-specific approaches to reform. This can be argued to be the case for China [65], but also in health systems development [4]. Areas such as global health and international development, which are increasingly framing key questions in terms of complex and adaptive systems, face questions around the institutions needed to foster innovation and maintain adaption for the benefit of a hoped-for future, emergent, state, whether in economic or market development or in health or social policy. Despite the limitations of the Chinese case, it should be considered a reference point with relevance for policy makers and policy-focused researchers elsewhere for understanding processes of managing for emergence in complex systems.

How to ensure that incentives create beneficial innovations or ensure the desired direction of reforms, and how to develop risk-tolerant implementation cultures [31] are substantial challenges in this kind of approach. Alongside the fostering of innovation, screening and evaluation present substantial challenges in complex systems. How to create mechanisms that can generate credible information for policy makers and implementers about the impacts of interventions where change is rapid, where starting points of most implementing jurisdictions vary in unknowable ways, and where interactions are complex, is a formidable task, though one in which China has some experience [66]. This remains under-theorised but is a necessary part of the experimenters’ toolkit [67].

## Abbreviations

CAS: Complex adaptive system
LMIC: Low- and middle-income countries
MDG: Millennium Development Goal(s)
NCMS: New Cooperative Medical Scheme

## Footnotes

1 Some constraints, such as absorptive capacity or exchange rate distortions due to aid inflows, are characteristic of aid programmes, and not solely constraints linked to health interventions [7].

2 Various typologies exist for classifying systems as simple, complicated, complex (and sometimes chaotic) [20, 79].

3 On fostering innovation in public services see [61] and in health services [32].

4 Wild et al. discuss the dangers of isomorphic mimicry, particularly in aid-dependent settings, where there is pressure to adopt supposed best practices, independent of their fit with local context.

5 Use of the term ‘positive deviance’ in development studies originates with research on nutrition practices in poor communities in Vietnam [74].

6 There are five tiers of government below the centre: provinces and municipalities; prefecture-level cities; counties and county-level cities; townships and towns; and villages and/or street-level urban jurisdictions.

7 Counties and county-level cities are the lowest level of administration with the full complement of government agencies, reflecting central ministries and other agencies. China has almost 3000 county-level administrative jurisdictions.

8 Two examples from Wu Yi, then Vice Premier of China, in relation to development of rural health insurance, a national policy process initiated in the early 2000s, illustrate this use: “the difference in conditions between different rural areas is very large, and it is impossible to adopt a one size fits all policy; the main thing is for each area to experiment in the process of implementation in line with local conditions” [80], and “We use a method in which the centre sets the principles of policy and localities concretely organise implementable policy; we encourage each locality to experiment and innovate” [81].

9 Fieldwork carried out by the author on county-level policy innovation in the development of China’s rural health insurance system found common rationalisations of policy flexibility and leeway in implementation between central rhetoric and sub-national governments (province, city and county).

10 China’s administration is split between rural and urban areas. Urban benefit systems have consistently been more generous than rural ones.
